# Unusual presentation of a five‐month‐old boy with NaPi2a homozygous mutation without hyperphosphaturia: Case report and review of the literature

**DOI:** 10.1002/ccr3.4740

**Published:** 2021-09-12

**Authors:** Renata Yakubov, Asaly Ayman, Adi Klein Kremer, An Bael, Machiel van den Akker

**Affiliations:** ^1^ Department of Pediatrics Hillel Yaffe Medical Center Hadera Israel; ^2^ Nephrology Unit Hillel Yaffe Medical Center Hadera Israel; ^3^ Department of Pediatrics ZNA Queen Paola Children’s Hospital Antwerp Belgium; ^4^ Pediatric Nephrology ZNA Queen Paola Children’s Hospital Antwerp Belgium; ^5^ Faculty of Medicine University of Antwerp Antwerp Belgium; ^6^ Department of Pediatric Hematology Oncology UZ Brussel Brussels Belgium

**Keywords:** case report, hypercalciuria, *NaPi2a* gene, nephrocalcinosis, phosphaturia, *SLC34A* gene

## Abstract

Deletions of the *NaPi2a* gene and mutations in the *SLC34A* gene should be considered in patients with atypical presentation, without phosphaturia, with mild hypo to normal phosphatemia, and nephrocalcinosis.

## Graphical abstract

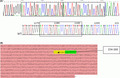

## 1 INTRODUCTION

We describe an infant with a *NaPi2a* homozygous mutation without hyperphosphaturia, with slightly lower to normal blood levels of phosphate, and with medullary nephrocalcinosis. Medullary calcinosis, with low‐normal blood level of phosphate, was not known to be associated with mutations in the *SLC34A* gene which encode the NaPi2a cotransporter in humans. When in infancy for evaluation reasons, an abdominal ultrasound is done, nephrocalcinosis is sometimes detected.^1^ When additional investigations reveal hypercalcemia, hypercalciuria, and without hyperphosphaturia, the diagnostic process can be challenging. Although the knowledge in regard to the calcium phosphate homeostasis is growing, partly due to the fast‐growing recognition of the role of genetic factors in the causation of many disorders, findings are not always consistent with the current medical literature.

The main regulators of calcium and phosphate hemostasis are the parathyroid hormones (PTH), calcitonin, 1,25‐dihydroxyvitamin D, and fibroblast growth factor 23 (FGF23). They coordinate the release of phosphate and calcium from the skeleton, their absorption from the food in the intestine and their reabsorption/excretion in the kidney. When hypercalcemic condition exists, the thyroid gland increases the secretion of calcitonin into the blood, while at the same time reduces the secretion of PTH. Calcitonin stimulates the osteoblasts to remove calcium form the blood. The low concentration of PTH inhibits removal of calcium from the bone and inhibits the formation of 1,25‐dihydroxyvitamin D (from vitamin D_3_) by the kidneys, increases the loss of calcium in the urine and inhibits the loss of phosphate in the urine. The latter can be explained by preserving the NaPi cotransporters in the apical membrane of renal proximal tubule cells, preventing phosphaturia.^2^ Low concentration of 1,25‐dihydroxyvitamin D will inhibit the absorption of calcium by the enterocytes of the duodenum, as well as the release of calcium into the blood by the osteoclasts.

Fibroblast growth factor 23 is a phosphaturic hormone produced by osteocytes and osteoblasts, which binds to FGF receptors in the presence of the transmembrane protein αKlotho. FGF23 mainly targets the renal proximal tubule to inhibit 1,25‐dihydroxyvitamin D production by altering the vitamin D–metabolizing enzymes CYP27b1 and CYP24a1 and to inhibit the expression of the sodium/phosphate cotransporters, NaPi2a and NaPi2c, thus inhibiting renal phosphate reabsorption.^3^ Deletions of the *NaPi2a* gene result mostly in phosphaturia, clear hypophosphatemia, elevated 1,25 (OH)_2_ vitamin D_3_ levels, hypercalcemia, and hypercalciuria. We describe an infant with an unusual presentation of NaPi2a homozygous mutation without hyperphosphaturia.

### 1.1 CASE REPORT

A five‐month‐old boy was admitted to the hospital after several weeks of vomiting, watery diarrhea (2–4x/day), and failure to thrive. Presence of polyuria and polydipsia. No fever was reported. Received oral vitamin D 400 IU daily up to the age of four months. The boy was born at 36 weeks of gestation by uncomplicated cesarean delivery after a pregnancy complicated by polyhydramnios, first noted at week 23 of gestation. Birth weight 3620 g (standard deviation (SD) 0.5), length 50 cm (SD 0.1), and head circumference 34 cm (SD −0.4). Physical examination was reported as normal. He was the third child of second‐degree consanguineous Israeli‐Arab parents. The family history shows that a paternal uncle has undergone a nephrectomy in infancy for unknown reason. At admission, he presented in stable condition with vital signs appropriate for his age (pulse 128/minute, blood pressure 90/50 mmHg, respiratory rate 30/minute, and normal peripheral perfusion) and normal hydration status. Weight 5.8 kg (SD −2.3), length 62 cm (SD −0.9), and head circumference 41.5 cm (SD −0.9). His physical examination was unremarkable.

Suspected of having cow's milk protein allergy, his formula was changed to hydrolyzed milk protein formula (Nutramigen A+), with good response. No medication, like phosphate supplementation, was given.

**FIGURE 1:**
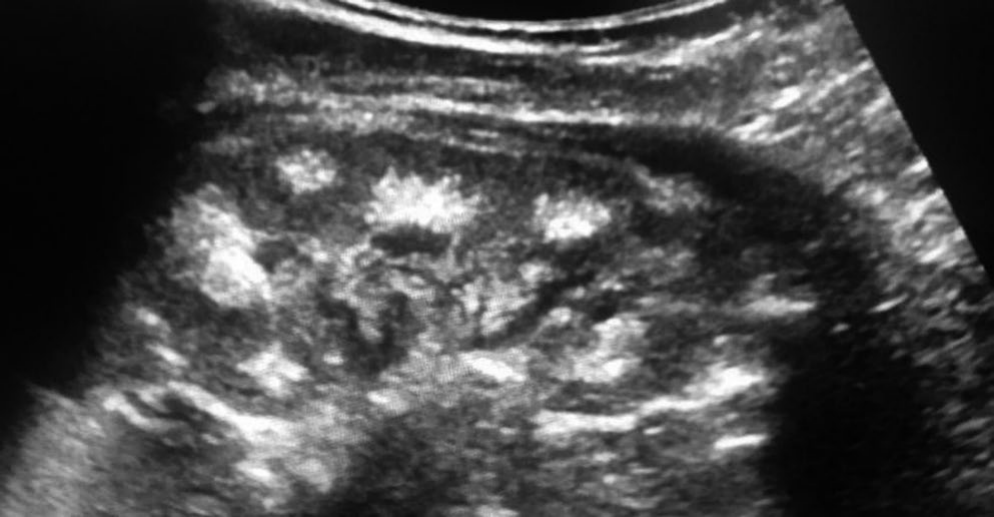
US image of the right kidney showing grade III medullary nephrocalcinosis and a mild hydronephrosis

Laboratory tests revealed hypercalcemia, hypercalciuria, and mildly low phosphatemia, in the presence of low values of PTH and normal values of 1,25‐dihydroxyvitamin D. The serum creatinine level is mildly elevated (see Table 1). The symptoms could be explained by the hypercalcemia, but the underlying cause is unclear. Renal ultrasound revealed grade III^1^ medullary nephrocalcinosis and a mild right hydronephrosis (see Figure 1). Treatment with oral tripotassium citrate was initiated in order to prevent formation of stones. After several days, oral chlorothiazide (2.5 mg × 1/day) was added. Additional tests were done to exclude and evaluate the possibility of Fanconi syndrome as a cause for hypercalcemia, hypercalciuria, and medullary nephrocalcinosis. The urine test revealed normal results for the total tubular reabsorption of phosphate and uric acid/creatinine ratio, while calcium/creatinine ratio and urine low molecular weight proteins were clearly elevated (see Table 1). Blood gas and glucose were normal. Fanconi syndrome was unlikely.

**TABLE 1:** Laboratory results

Test	Results				Range normal values
Age	5 months	7 months	14 months	18 Months	
Blood					
Calcium (mg/dl)	11.7	12	11	11	8.0–10.7 mg/dl
Phosphate (mg/dl)	4	4.6	4.5	4.4	4.5–6.5 mg/dl
Sodium (mmol/L)	142	144	138	137	135–148 mmol/L
Potassium (mmol/L)	4.6	4.9	4.8	4.5	3.5–5.6 mmol/L
Alkaline Phosphatase (U/L)	260	248	253	240	122–469 U/L
Creatinine (mg/dl)	0.37	0.38	0.44	0.49	0.17–0.35 mg/dl
Uric Acid (mg/dl)	4.6	4.2	3.4	4.7	3,4–7,0 mg/dl
Magnesium (mg/dl)	2.3	2.4	2.3	2.1	1,7–2,3 mg/dl
Parathyroid hormone (pg/ml)	1.2		2.5	3.9	15–65 pg/ml
25‐Hydroxyvitamin D (pg/ml)	53	40	34	34	35–75 pg/ml
1,25‐Dihydroxy vitamin D (1,25(OH)2D) (pg/ml)			73	75	20–100 pg/ml
Blood gas (capillary)					
Ph	7.44			7.41	7.35–7.45
HCO3(mmol/L)	22.5			25.7	22–26 mmol/L
Urine					
Calcium/ creatinine ratio (mg/mg)	1.45	1.36	1.9	1.3	0–0.5 y: 0.2–0.6 mg/mg
					0.5–1.0 y: 0.2–0.4 mg/mg
					>1.0 y: <0.2 mg/mg
Protein/creatinine ratio (mg/mg)	0.54	0.51	0.72	0.3	0–0.5 y: 0–0.5 mg/mg
					>0.5 y: 0–0.2 mg/mg
Low molecular weight proteins (mcg/L)	297				<200 mcg/L
Uric acid/creatinine ratio (mg/mg)	1.52	1.1	1.3	1	0.5–1 mg/mg
Albumin/creatinine ratio (mg/g)		56	29	25	0–30 mg/g
Total reabsorption of phosphate (TRP) (%)	96%	90%	95%	96%	>85%

**FIGURE 2:**
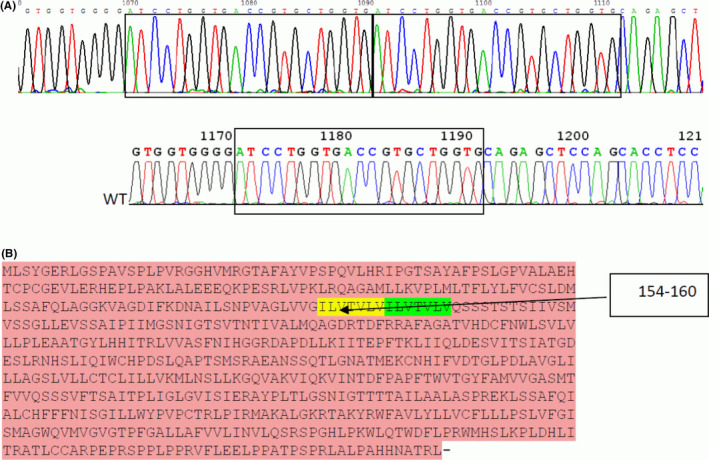
Genomic DNA sequence analysis of *SLC34A1*. Panel A reveals a homozygous inserted duplication of 21 bp (ATCCTGGTGACCGTGCTGGTG) in exon 5 at positions g.1170_1190 causing a duplication of amino acids (ILVTVLV) at positions 154 to 160 of the NaPi2a protein (panel B). The bottom row shows a healthy control with the wild‐type (WT) sequence

In further course the 1,25‐dihydroxy vitamin D remained in the normal range whereas the PTH remained very low. Considering hypercalcemia, hypercalciuria, low to normal phosphate, low level of PTH, and the inappropriate normal level of 1,25‐dihydroxy vitamin D, gene sequencing of *CLCN5*/*OCRL*/*CYP24A1* genes was done. The results were normal, ruling out Dent disease and a loss‐of‐function mutations in 24 alpha hydroxylase. Furthermore, sequence analysis of the *SLC34A1* gene, which encodes for the renal NaPi2a cotransporter, was performed, revealing a known homozygous loss‐of‐function mutation in the NaPi2a protein (see Figure 2).

Over the time, he developed normally. At the age of 15 month, his physical examination was normal. Weight 10.5 kg (SD 0.2), length 79 cm (SD 0.5), and head circumference 47 cm (SD 0.1). He is regularly seen by the nephrologist in the outpatient clinic.

## 2 DISCUSSION

We describe an infant with a homozygous duplication in *SLC34A* gene with medullary nephrocalcinosis, but without hyperphosphaturia, and with slightly low to normal blood levels of phosphate. To the best of our knowledge, this is the first publication, reporting these findings.

Hypophosphatemia is critical in FGF23 inhibition because of negative feedback, which causes eventually hypercalcemia, hypercalciuria, and medullary nephrocalcinosis. The almost normal phosphatemia in our patient may be explained by a yet unknown aspect of the mutation or due to the nephrocalcinosis causing renal failure with reduced GFR resulting in pseudo‐normophosphatemia.

Maintenance of phosphate in the normal range is critical for many biologic reactions. About 99% of the phosphate in the body is intracellular and only one percent is extracellular. The absorption of phosphate across the cell membrane happens by sodium‐dependent (Na), inorganic phosphate (Pi) transporter (NPT) proteins. Phosphate in the gastrointestinal tract is absorbed at the brush border by the NaPi2b cotransporter, which is encoded by the *SLC34A2* (solute carrier family 34, member 2) on chromosome 4 (4p15.2). In the proximal tubule of the kidneys two cotransporters, NaPi2a and NaPi2c, encoded by *SLC34A1* (on chromosome 5 (5q35.3)) and *SLC34A3* (on chromosome 9 (9q34.3)) genes respectively,^4^ are responsible for reabsorption of 70%–80% of filtered phosphate.^5^, ^6^

Up to date, the literature is lacking clinical and laboratory consequences of defect in the NaPi2a cotransporter in humans, and most of the current descriptions are based on mice models,^7^ whereas there are large clinical descriptions of NaPi2c cotransporter defects and their consequences in humans.^8^ Sequence variants in *SLC34A1* have been reported as potential causes of renal phosphate leak in the heterozygous state in human subjects. However, subsequent studies have not confirmed the functional effects of these polymorphisms.^9^, ^10^ In a more recent study, Lapointe et al.^11^ identified several heterozygous *SLC34A1* sequence variants in association with renal phosphate leak and hypercalciuria. However, functional analysis of these variants eventually also ruled out their role in renal phosphate leak due to messenger RNA (mRNA) expression in *Xenopuslaevis* oocytes. In contrast, homozygous loss‐of‐function mutations of the *SLC34A3* gene, which encodes NaPi2c, have been shown to cause hereditary hypophosphatemic rickets with hypercalciuria (HHRH).^8^, ^12^

Magen et al.^13^ report two siblings with autosomal recessive Fanconi's syndrome and hypophosphatemic rickets (Ca 9.5 (9.1–9.9) mg/dl, P 2.1 (2.0–2.2) mg/dl, TRP 57 (49–66) %) caused by a homozygous in‐frame duplication of 21 bp in *SLC34A1*, which encodes the renal sodium‐inorganic phosphate cotransporter NaPi2a. Functional studies of mutant NaPi2a in Xenopus oocytes and opossum kidney cells demonstrated failure of the transporter to reach the plasma membrane and loss of phosphate transport activity. Schlingmann et al.^14^ describe four patients from three consanguineous families with typical IIH (idiopathic infantile hypercalcemia) with renal phosphate wasting and symptomatic hypercalcemia (Ca 12.7 (11.6–14) mg/dl, P 2.8 (1.5–4.5) mg/dl), at which autosomal recessive mutations in the *SLC34A1* gene were identified. Next, the *SLC34A1* gene was screened in a larger cohort of 126 sporadic IIH patients. Biallelic mutations were identified in 11 patients. In total, 16 different mutations were identified.

Here we report an infant with a similar mutation in the *SLC34A* gene, but presenting with low‐normal levels of phosphate without phosphaturia (Ca 11.7 mg/dl, P 4 mg/dl, TRP 96%).

To date, mutations in the *SLC34A* gene which encode the NaPi2a cotransporter have not been found to cause medullary calcinosis in the presence of normal blood level of phosphate in humans. Most patients with a known autosomal recessive homozygous duplication in *SLC34A* gene have been found to have hypercalcemia, hypercalciuria, clear hypophosphatemia, medullary nephrocalcinosis, and rickets. Demir et al.^15^ report two children who present with idiopathic infantile hypercalcemia (without hypercalciuria and rickets), secondary to the loss‐of‐function mutation in NaPi2a (pI154_V160dub). There are no reports published of patients with mutated Napi2a with low‐normal to normal blood levels of phosphate and without phosphaturia.

NaPi2a cotransporter knock out in mice exhibit increased urinary phosphate excretion, clear hypophosphatemia, and an appropriate elevation of serum 1,25‐dihydroxyvitamin D with attendant hypercalcemia, hypercalciuria, and decreased PTH levels.^7^ In humans with NaPi2c defect, the same features can be observed. It can be distinguished from the X‐linked hypophosphatemic rickets by the elevated 1,25‐dihydroxyvitamin D and the normal or low‐normal fibroblast growth factor 23 (FGF 23) levels.^8^, ^12^

## 3 CONCLUSION

In our child, a known homozygous loss‐of‐function mutation in the NaPi2a protein has been found, despite absence of phosphaturia. Our child has a slightly lower to normal phosphate level in the presence of depressed level of PTH and inappropriate normal 1,25‐dihydroxyvitamin D, these findings are inconsistent with what is known to date in the medical literature. Deletions of the *NaPi2a* gene and mutations in the *SLC34A* gene should be considered in patients with atypical presentation, without phosphaturia, with mild hypo to normal phosphatemia, and nephrocalcinosis.

We need to request *SLC34A* gene sequencing in patients with medullary nephrocalcinosis even if phosphate levels are found to be normal or mildly lower, and we need to investigate further the exact function of NaPi2a cotransporter.

## 4 ETHICS APPROVAL AND CONSENT TO PARTICIPATE

Not applicable.

## CONSENT FOR PUBLICATION

Written consent was obtained from the patient´s legal guardian(s) of this case report and any accompanying images. A copy of the written consent is available for review by the editor‐in‐chief of this journal.

## CONFLICT OF INTEREST

All authors declare no conflict of interest.

## AUTHORS’ CONTRIBUTIONS

RY was responsible for the data collection, obtaining consent, and the author of the manuscript. AA was responsible for part of the discussion section. AKK was responsible for part of the discussion and reviewing the paper. AB was responsible for part of the discussion and reviewing the paper. MA was responsible for the writing and finalizing of the paper. All authors read and approved the final manuscript.

## Data Availability

All data are available in our hospital.
